# Impact of Dentin Pre-treatment Strategies on Colour Stability of Nanohybrid Composite Resin: An In Vitro Spectrophotometric Analysis

**DOI:** 10.7759/cureus.90169

**Published:** 2025-08-15

**Authors:** Harsha Nandhini Doraiswamy, Arasappan Rajakumaran, Seshan Rakkesh R, Mathan Rajan Rajendran

**Affiliations:** 1 Conservative Dentistry and Endodontics, Sri Ramachandra Dental College and Hospital, Chennai, IND

**Keywords:** 2% chlorhexidine, collagen cross-linkers, colour stability, dentin pre-treatment, mmp inhibitors, riboflavin, spectrophotometer

## Abstract

Introduction: Matrix metalloproteinases (MMPs) are endogenous dentinal enzymes that remain latent until activated in acidic environments. During self-etch or etch-and-rinse adhesive procedures, activated MMPs degrade the dentin matrix, breaking down type I collagen fibres and thereby compromising the hybrid layer and bond strength. To mitigate this degradation, MMP inhibitors are employed as dentin pre-treatment agents, effectively limiting collagen fibre degradation and enhancing bond strength. MMP inhibitors help preserve the integrity of the adhesive interface between the resin composite and dentin. Chlorhexidine (CHX), a widely used MMP inhibitor, is associated with a potential adverse effect of dental staining. A recently introduced collagen crosslinker, riboflavin, shows promising potential for enhancing bond strength. However, the influence of riboflavin on the colour stability of composite resin has not been evaluated.

Aim: To evaluate the effect of various dentin pre-treatment strategies on the colour stability of nanohybrid composite resin.

Methods: Thirty* *extracted permanent molars were collected and subjected to thorough scaling and pumice polishing. The teeth were stored in 0.1% thymol until further use. Thirty mid-coronal dentin discs were prepared from the specimens. Each sample was etched with 37% phosphoric acid for 15 s, rinsed with water, and blot-dried. The samples were randomly divided into three groups, with 10 specimens in each group. Group I (control) received no pretreatment prior to bonding. Group II involved the application of 2% CHX gel (Chlorex Gel, Waldent, New Delhi, India) for 60 s, followed by blot drying. In group III, a 0.1% riboflavin solution was prepared by dissolving a 10 mg tablet in distilled water and applied to the dentin surface, followed by irradiation with blue light-emitting diode light for two minutes, maintaining a distance of 1 mm from the dentin disc. Subsequently, two thin layers of fifth-generation bonding agent (Meta-P&Bond, Meta Biomed Co., Ltd., Chungcheongbuk-do, South Korea) were applied and light-cured for 20 s, followed by placement of a nanohybrid composite resin (Beautifil II, Shofu Inc., Kyoto, Japan). The baseline colour change was measured in the spectrophotometer (CM-5, Konica Minolta, Tokyo, Japan). Subsequently, the samples were subjected to 1,000 thermal cycles in a thermocycler to simulate six months of ageing. The immediate colour change and the colour change after thermocycling were measured using spectrophotometric analysis. The colour change was assessed using the CIELAB colour system, and the ΔE* values were calculated.

Results: The Kolmogorov-Smirnov test was conducted to evaluate the normality of the data, followed by one-way ANOVA and Tukey’s Post-hoc test for multiple pairwise comparisons. The highest mean discolouration was observed in group II (CHX), with a value of 4.09, followed by group III, with a ΔE* value of 3.91. The lowest mean discolouration was observed in group I, with a value of 2.69. Pairwise comparison revealed a statistically significant difference between groups I and II.

Conclusion: Preserving bond strength without compromising aesthetic outcome is crucial in restorative dentistry. Riboflavin, a collagen crosslinking agent, emerges as a promising alternative. Its potential to stabilise the dentin collagen matrix may enhance the durability of adhesive interfaces while maintaining the optical properties necessary for aesthetic restorations.

## Introduction

Dentin is an intrinsically hydrated tissue. The intertubular dentin contains collagen fibrils exhibiting the characteristic collagen banding patterns [1]. Intertubular dentin is transversed by submicron channels that facilitate the movement of tubular fluid and fibrillar structures between adjacent tubules. Bonding to dentin is unpredictable owing to its high organic content, primarily type I collagen fibres, which are denatured by the phosphoric acid during etching [1]. The smear layer formed during cavity preparation is removed by low pH etchants, which may activate the inherent matrix metalloproteinases (MMPs) within the dentin, leading to collagen degradation and a consequent reduction in bond strength [2].

Dentin pretreatment strategies aimed at enhancing bond strength include the use of MMP inhibitors and collagen crosslinkers. MMP inhibitors act antagonistically to MMPs, protecting collagen fibres within the hybrid layer from enzymatic degradation and thereby maintaining the integrity and durability of the adhesive bond. They also contribute to a decrease in interfacial nano-leakage [3]. Commonly used MMP inhibitors include chlorhexidine (CHX), tetracyclines and their derivatives, quaternary ammonium compounds, benzalkonium chloride, galardin, and ethylenediaminetetraacetic acid [3].

Collagen crosslinkers protect collagen fibrils from further degradation by enhancing the chemical and mechanical properties of the collagen matrix. The application of extrinsic collagen crosslinking agents promotes the formation of inter- and intra-molecular crosslinks. Selective crosslinkers increase the ultimate tensile strength and elastic modulus of demineralised dentin. Common collagen crosslinkers include proanthocyanidins, epigallocatechin-3-gallate, riboflavin, glutaraldehyde, and carbodiimides [4].

Exposure of riboflavin to blue light generates reactive oxygen species that promote crosslinking within the collagen network, enhancing fibril stiffness. In addition, this process limits protease activity, safeguards telopeptides by inhibiting collagenases and inactivating C-terminal telopeptides, and binds to critical peptide bonds, thereby fortifying the dentin matrix and ensuring a stable substrate for subsequent adhesive application [5,6]. A recent meta-analysis shows that riboflavin, a collagen crosslinker, demonstrates superior performance better than that of CHX, an MMP inhibitor [7]. When employed as dentin pre-treatment agents, MMP inhibitors should not produce discolouration of composite resin, as this might adversely influence the aesthetic outcome. Literature evidence suggests that 2% CHX produces discolouration when used as a pre-treatment strategy to enhance bond strength [8,9].

Riboflavin, a photoactivated collagen crosslinker, has been explored as a dentin pretreatment strategy to improve bond durability. However, its influence on the colour stability of nanohybrid composite resin has not been fully established. The aim of this study was to compare the effects of 2% chlorhexidine and 0.1% riboflavin dentin pretreatment with a negative control (no pretreatment) on the colour stability of nanohybrid composite resin, measured immediately and after thermocycling using spectrophotometric analysis. The objective was to quantify colour changes using spectrophotometric evaluation based on the CIELAB system.

## Materials and methods

The research protocol was approved by the clinical research ethics committee of the university (CSP/24/DEC/154/441). The required sample size was determined using G*Power 3 software (Heinrich Heine University Düsseldorf, Düsseldorf, Germany). The sample size was calculated based on the effect size reported in the study by Iskander et al. (2015) [8]. Based on the F test family, with an effect size of 0.62, an alpha error of 0.05, and a power of 0.8, the minimum sample size was calculated to be 30. Thirty extracted permanent mandibular molars were included in the study, with 10 specimens allocated to each group. Teeth exhibiting dental caries, gross structural decay, or pre-existing restorations were excluded. The extracted molars were collected and subjected to thorough scaling followed by polishing with pumice. Thereafter, the teeth were stored in 0.1% thymol solution until further use. Thirty mid-coronal dentin discs, each measuring 1.5 mm in thickness, were sectioned from the specimens using a saw microtome under water cooling (Leica SP1600, Leica Microsystems GmbH, Wetzlar, Germany) and subsequently polished using a Bainpol VT polishing machine (Bainpol, Chennai, India). The dentin discs were etched with 37% phosphoric acid using a micro-applicator tip (Oro, Pune, India) for 15 seconds, rinsed thoroughly with water and blot-dried using an air-water syringe.

The discs were randomly allocated into three groups (n = 10) based on the dentin pre-treatment protocol. Group I (control): no pretreatment prior to the bonding protocol. Group II (CHX): application of 2% CHX gel (Chlorex Gel, Waldent, New Delhi, India) for 60 seconds, followed by blot drying. Group III (riboflavin): a 0.1% riboflavin solution was prepared by dissolving a 10 mg tablet in distilled water and applied to the dentin surface. This was followed by irradiation with Woodpecker D LED unit (Guangxi, China) operating at a wavelength of 1,000 mW/cm^2^ for two minutes, maintaining a distance of 1 mm from the dentin disc to induce the excited triplet state and generate reactive oxygen species that promote collagen crosslinking.

**Figure 1:**
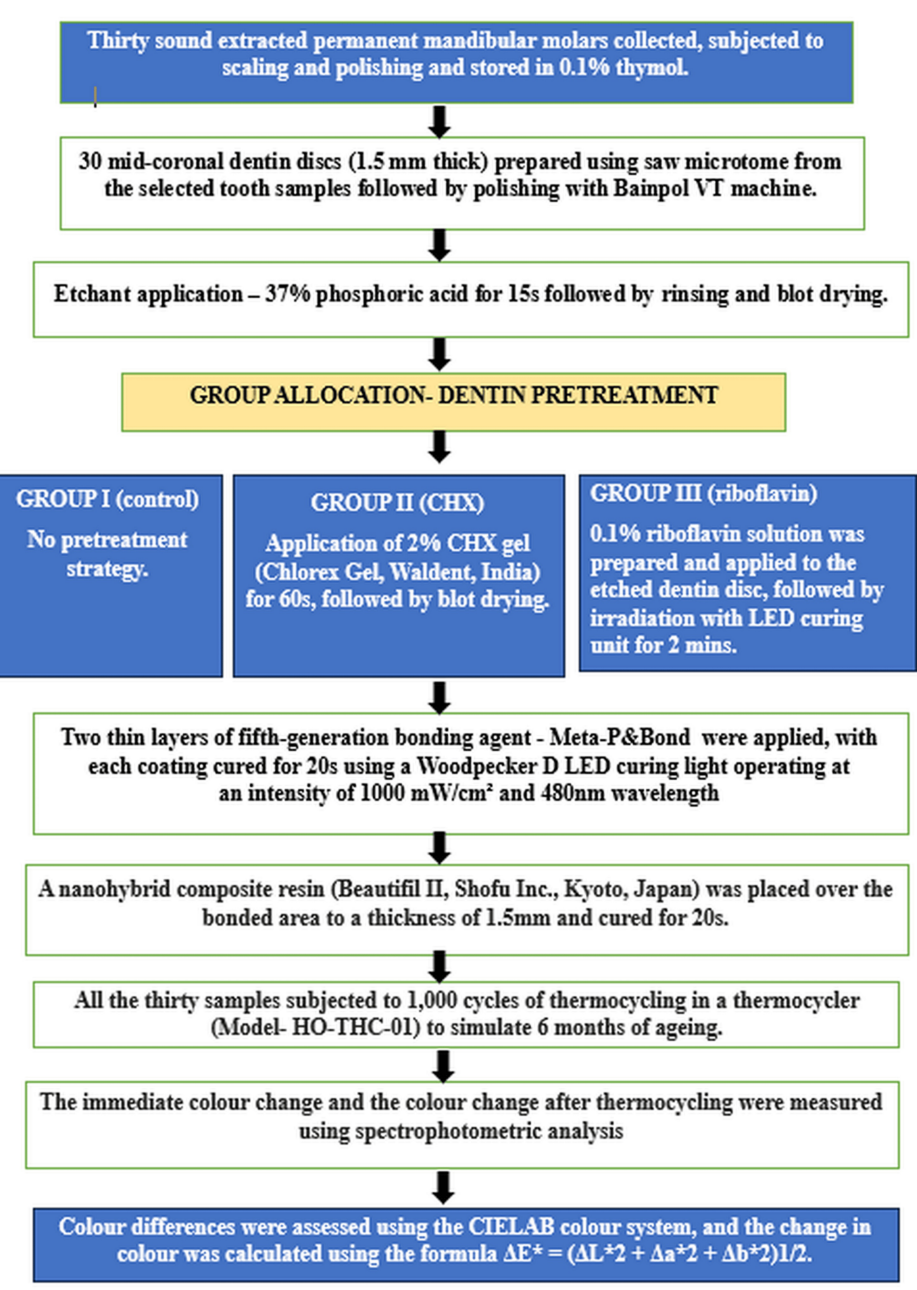
Flowchart of study methodology. Flowchart depicting the step-by-step methodology for sample preparation, dentin pretreatment, bonding, nanohybrid composite resin placement, thermocycling, and measurement of colour change in the present study. CHX: chlorhexidine; LED: light-emitting diode.

**Figure 2:**
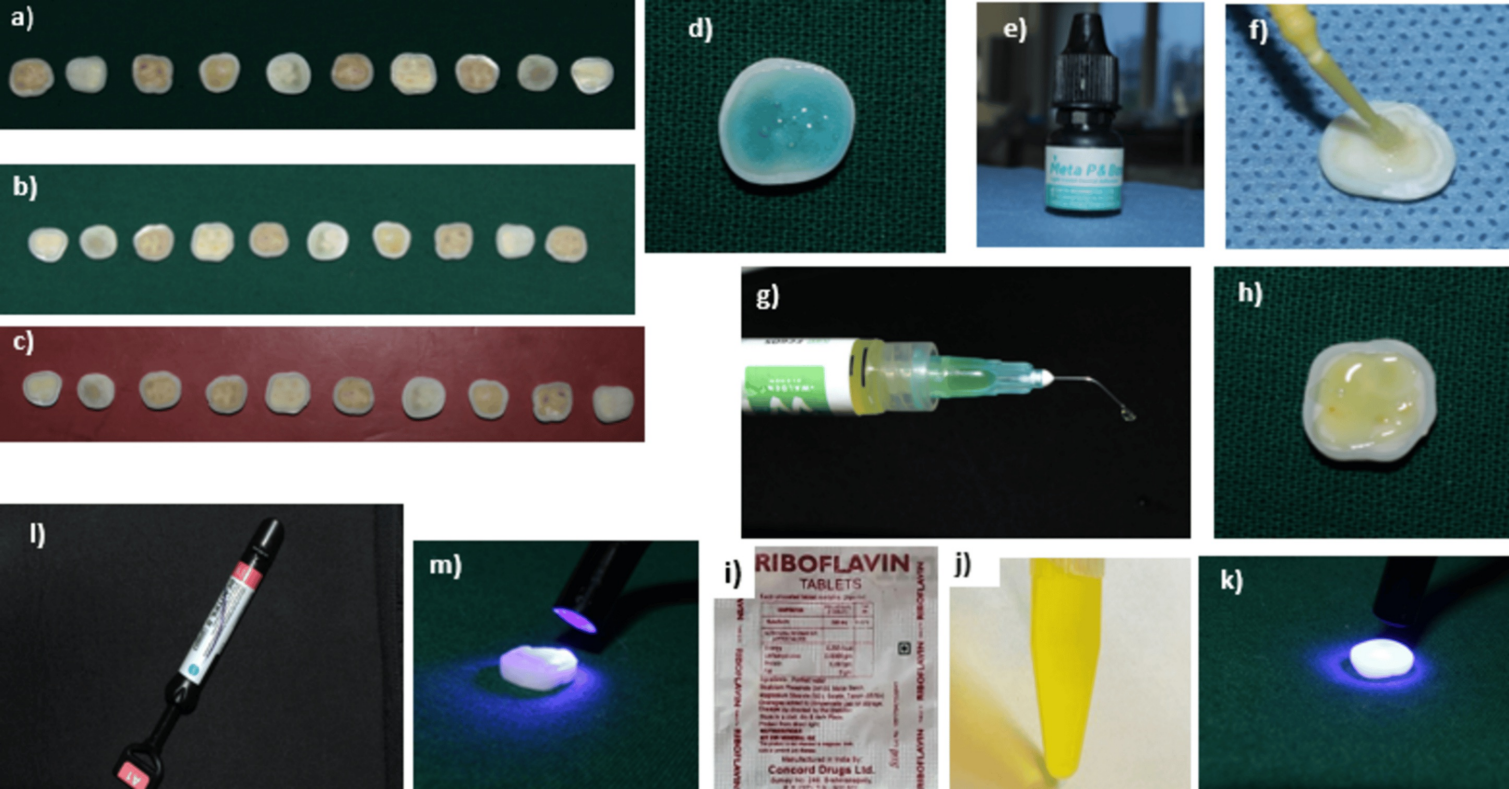
Visual representation of experimental workflow. (a-c) Prepared dentin discs for group I (control), group II (2% chlorhexidine), and group III (0.1% riboflavin), respectively. (d) Etching dentin disc with 37% phosphoric acid. (e) Fifth-generation bonding agent (Meta-P&Bond). (f) Application of the bonding agent onto the dentin surface. (g) 2% chlorhexidine gel. (h) Application of 2% chlorhexidine gel to the dentin surface. (i) Riboflavin tablet used for solution preparation. (j) Prepared 0.1% riboflavin solution. (k) Irradiation of riboflavin-treated dentin surface with light-emitting diode (LED) curing unit. (l) Nanohybrid composite resin (Beautifil II). (m) Light curing of composite resin using LED curing unit.

**Figure 3:**
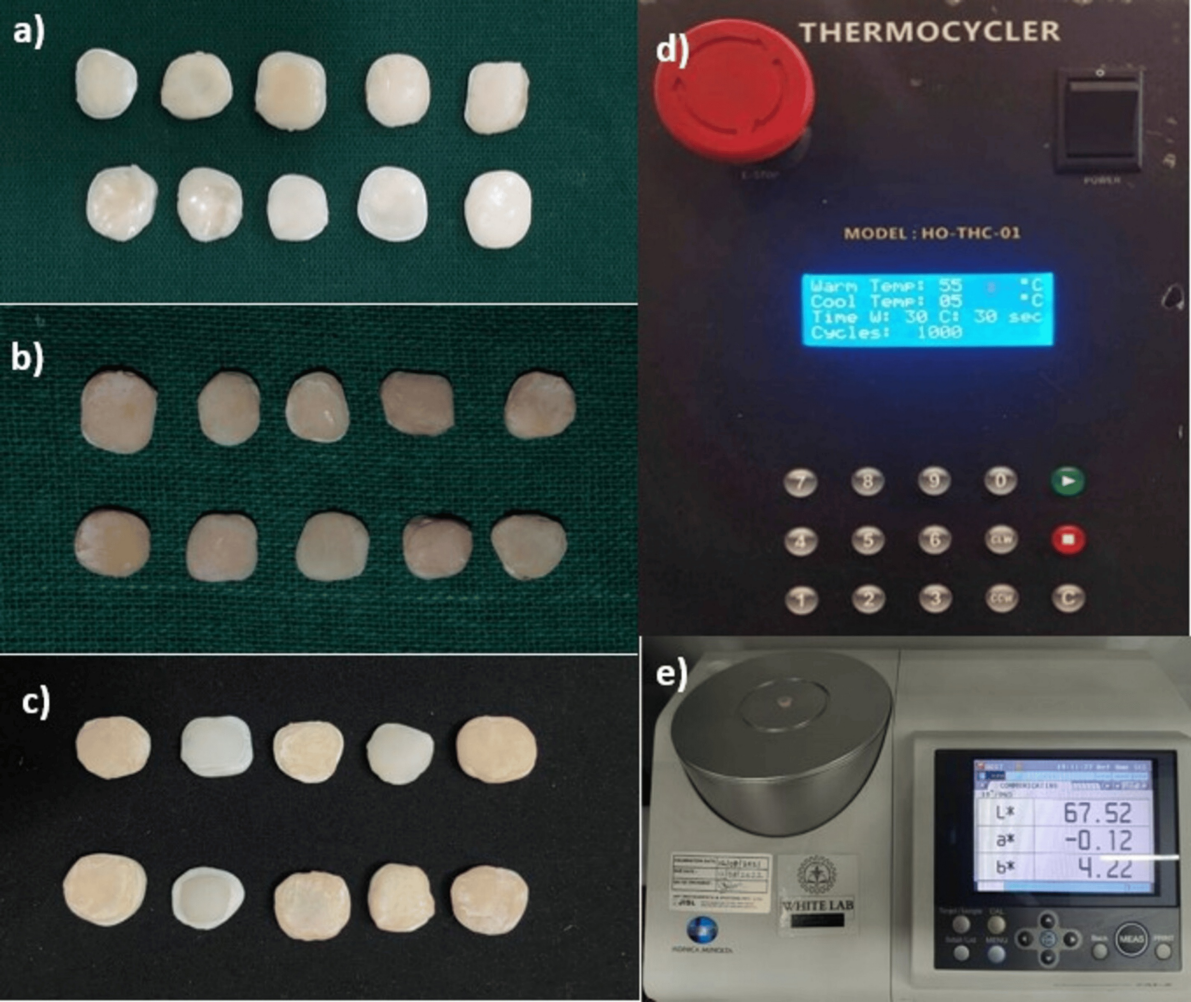
Colour change observed in the individual groups following thermocycling. (a) Group I (control). (b) Group II specimens treated with 2% chlorhexidine (CHX). (c) Group III specimens treated with 0.1% riboflavin. (d) Holmarc Thermocycler Model HO-THC-01 used for ageing simulation. (e) Spectrophotometer (Konica Minolta CM-5) employed for colour change evaluation.

Following dentin pretreatment, two thin layers of a fifth-generation bonding agent (Meta-P&Bond, Meta Biomed Co., Ltd., Chungcheongbuk-do, South Korea) were applied, with each coating cured for 20 seconds using a Woodpecker D LED curing light operating at an intensity of 1000 mW/cm² and 480 nm wavelength. A nanohybrid composite resin (Beautifil II, Shofu Inc., Kyoto, Japan) was placed over the bonded area to a thickness of 1.5 mm using the Optrasculpt posterior composite modelling instrument (Ivoclar Vivadent AG, Schaan, Liechtenstein). The immediate colour change was recorded using a spectrophotometer (CM-5, Konica Minolta, Tokyo, Japan) prior to subjecting the samples to thermocycling. Subsequently, the samples were subjected to 1,000 cycles in a thermocycler (Model HO-THC-01, Holmarc, Kochi, India) to simulate six months of intraoral ageing. The apparatus, equipped with stainless steel hot and cold water baths, maintains temperatures between 5°C and 60°C with a dwell time of 30 seconds in each bath (programmable within a range of 0-999 seconds). A stainless steel basket holds the samples and is moved between the baths via a pivoting arm mechanism powered by a stepper motor. The colour change after thermocycling was also measured using spectrophotometric analysis (CM5). Colour differences were assessed using the CIELAB colour system, and the change in colour was calculated using the formula ΔE* = (ΔL*2 + Δa*2 + Δb*2)1/2. A ΔE* value of 3.3 is considered clinically perceptible. The CIELAB colour model defines colour using three parameters: L represents lightness, ranging from 0 (black) to 100 (white); a indicates the shift along the green-red axis, with negative values toward green and positive values toward red; and b* describes the variation along the blue-yellow axis, where negative values represent blue and positive values represent yellow [10]. The schematic representations of the experimental methodology are provided in Figures 1, 2. The colour change after thermocycling is depicted in Figure 3.

Statistical analysis

The normality of the data was evaluated using the Kolmogorov-Smirnov test. As the data followed a normal distribution, parametric statistical methods were applied. Differences in colour change among the three groups were assessed using one-way analysis of variance (ANOVA). When statistically significant differences were observed, Tukey’s post hoc test was used to perform pairwise group comparisons. A p-value of <0.05 was considered statistically significant. Statistical analysis was performed using IBM SPSS Statistics for Windows, version 20.0 (IBM Corp., Armonk, NY).

## Results

**Figure 4:**
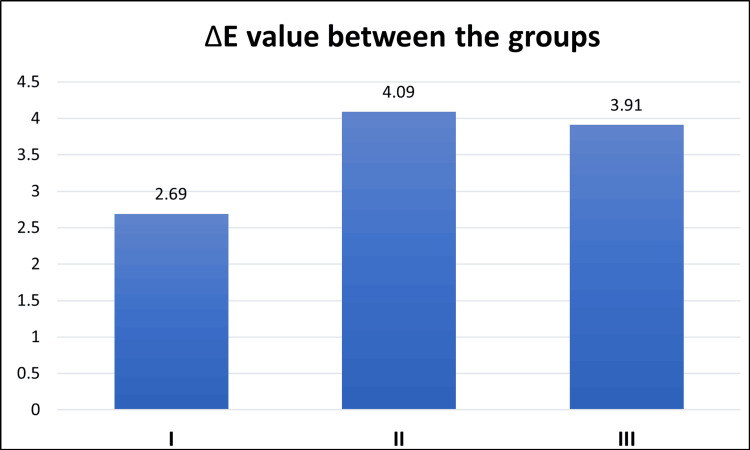
Bar chart showing colour change (ΔE).

To evaluate the distribution of the data, the Kolmogorov-Smirnov test was conducted, confirming that the data were normally distributed (Table 1). Consequently, parametric statistical methods were considered appropriate for analysis. Differences among the three groups were evaluated using one-way analysis of variance (ANOVA) (Table 2). Where significant differences were observed, Tukey’s post hoc test was employed to conduct multiple pairwise comparisons and identify specific intergroup differences (Table 3). The mean values of discolouration are presented in the bar graph (Figure 4). Group II (CHX) exhibited the highest mean discolouration value of 4.09, followed by group III (riboflavin) with a value of 3.91. The lowest mean value was observed in group I (control) at 2.69 (Figure 4 and Table 2). A statistically significant p-value of 0.018 was observed in the one-way ANOVA. Therefore, Tukey's post hoc pairwise comparison was conducted to analyse the significance between the individual groups. Group II (CHX) demonstrated significantly greater discolouration than that of group I, with a p-value of 0.024. No statistically significant difference was found between group I and group III (p = 0.053), and between group II and group III (p = 0.930).

**Table 1:** Assessment of data normality using Kolmogorov–Smirnov and Shapiro–Wilk tests. The normality of data distribution was assessed using the Kolmogorov–Smirnov and Shapiro–Wilk tests. A significance level of α = 0.05 was adopted for all statistical analyses, with * p > 0.05 indicating no significant deviation from normality and supporting the use of parametric tests.

Group	Kolmogorov-Smirnov			Shapiro-Wilk		
	Statistic	df	P-value	Statistic	df	P-value
I	0.206	10	0.200^*^	0.870	10	0.099
II	0.146	10	0.200^*^	0.942	10	0.578
III	0.210	10	0.200^*^	0.896	10	0.200

**Table 2:** One-way ANOVA for intergroup comparison of mean ΔE values. Mean values, standard deviations (SD), and 95% confidence intervals (CI) for each group. ANOVA test statistic (F) and corresponding p-value are reported. Asterisk (*) indicates a statistically significant difference between groups (p < 0.05).

Group	Mean	SD	95% confidence interval (CI) for mean		F	P-value
			Lower bound	Upper bound		
I	2.69	1.45	1.65	3.73	4.683	0.018*
II	4.09	1.02	3.35	4.82		
III	3.91	0.71	3.40	4.41		

**Table 3:** Pairwise comparison of group mean ΔE values using Tukey’s HSD post-hoc test. Mean differences and associated pairwise p-values were computed using Tukey’s honestly significant difference (HSD) post-hoc test. Statistical significance was set at p < 0.05. Asterisk (*) indicates a statistically significant difference. Std. error refers to the standard error of the mean difference between groups.

Group comparing	Group compared	Mean difference	Std. error	P-value	95% confidence interval	
					Lower bound	Upper bound
I	II	-1.39200^*^	0.49476	0.024*	-2.6187	-0.1653
	III	-1.21200	0.49476	0.053	-2.4387	0.0147
II	III	0.18000	0.49476	0.930	-1.0467	1.4067

Tests of normality show that the data are normally distributed; therefore, parametric tests are used for data assessment.

Mean value of discolouration

The mean discolouration was highest in group II, with a value of 4.09, followed by group III at 3.91. The lowest mean value was observed in group I at 2.69 (Table 2). Group II exhibited significantly greater discolouration than that of group I. This difference was statistically significant. However, no statistical significance was observed between groups I and III and between groups II and III (Table 3).

## Discussion

In this study, 2% CHX was used as one of the experimental groups. According to Kazemi-Yazdi et al. [11], who investigated the effect of various CHX concentrations on bond strength, 2% CHX shows superior performance to that of 0.2% in increasing microtensile bond strength [11,12]. Light-activated riboflavin generates oxygen free radicals, leading to new covalent bond formation between the hydroxyl functional groups of riboflavin and the amino acids proline or lysine in collagen [13]. This strengthens the collagen network, protecting it from MMP degradation. A 2021 systematic review and meta-analysis shows that CHX enhanced bond strength after ageing [14]. However, when various strategies were compared to enhance bond strength, riboflavin and carbodiimide demonstrated more effective alternatives than CHX [7].

Maintaining bond integrity without compromising aesthetics is essential for successful restorations. In the present study, a spectrophotometer was employed to evaluate colour changes to minimise subjective errors. The CIELAB colour system, currently regarded as one of the most accurate methods for evaluating colour variations, was employed. CIELAB (CIE L*a*b*) is a 3D colour space used to measure and compare all perceptible colours utilising three parameters. The colour change was calculated using the formula ΔE* = (ΔL*2 + Δa*2 + Δb*2)1/2 [10]. A colour change with ΔE <1 is generally imperceivable, values between 1 and 3.3 are detected by trained observers, and values > 3.3 could be detectable by laypersons [9,15]. Only the control group exhibited a ΔE value <3.3. The next closest value was observed in the riboflavin group (ΔE = 3.91), while the highest colour change value was observed in the CHX group (ΔE = 4.09) (Table 2). The composite shade used in the study was A1, a comparatively lighter shade used to measure the colour stability influence of MMP inhibitors on composite resin.

Chlorhexidine used as a dentin pretreatment has been associated with reduced polymerisation efficiency of resin composites, potentially leading to a higher concentration of residual monomers and compromising long-term colour stability [11]. The oxidation of CHX leads to a colour change, potentially owing to the reaction of oxygen with the free amino groups in CHX. In the biofilm, proteins react with sugars through the Maillard reaction, forming glycosylamines that are inherently unstable. These intermediates convert into ketosamines, a process accelerated by chlorhexidine (CHX). The reaction triggers polymerisation, ultimately producing brown pigments referred to as melanoidins [16]. In this study, we hypothesise that riboflavin competes with the photo initiators in the composite, thereby hindering the polymerisation reaction and leading to a higher number of residual monomers. Discolouration has been attributed to oxidative changes in the polymer matrix structure and the oxidation of the unreacted pendant methacrylate groups [17].

In this study, all specimens were subjected to thermocycling to simulate the intraoral environment and evaluate the actual discolouration potential of various experimental groups on the composite resin. In total, 1,000 thermocycles were performed using the Holmarc Thermocycler Model HO-THC-01, which corresponds to approximately six months of ageing. The total-etch strategy causes more collagen degradation than that of the self-etch approach [18]. Future studies should include different types of composites, a self-etch strategy, and explore a broad range of experimental dentin pre-treatment strategies.

The strengths of this study include the use of riboflavin, a recently validated collagen crosslinker supported by meta-analytic evidence, as a dentin pretreatment agent. By comparing its effects with chlorhexidine, the established gold standard, the study provides valuable insights into how different pretreatment protocols affect the colour stability of nanohybrid composite resins, which are widely used in aesthetic restorations. Furthermore, colour change assessment utilised quantitative methods, ensuring objective measurement beyond visual or qualitative evaluation. The objective quantification of colour change was performed using a benchtop spectrophotometer (CM-5), which, according to the manufacturer’s specifications, has a repeatability of ΔE*ab = 0.04 and an inter-instrument agreement of ΔE*ab = 0.15. The device was automatically calibrated at each startup using the internal white and 100% reference plate, ensuring measurement reliability [19].

Limitations

The absence of a self-etch adhesive strategy is a limitation, as its inclusion could have provided findings more representative of current clinical adhesive practices. Only one type of composite resin and adhesive system were tested, which may restrict the generalisability of the results. The relatively small sample size further limits statistical power. The ageing protocol involved short-term thermocycling (six months), which does not fully replicate the complexity of long-term intraoral conditions. As with all in-vitro studies, the results cannot be directly extrapolated to clinical performance. Additionally, variations in riboflavin solution preparation may influence its crosslinking efficacy, potentially introducing variability in the outcomes.

## Conclusions

Based on the findings of this study, which incorporated two different dentin pretreatment strategies, an MMP inhibitor and a collagen crosslinker, along with a negative control group, riboflavin (a collagen crosslinker) performed better than the MMP inhibitor, chlorhexidine, and was comparable to the performance observed in the negative control group. Maintaining bond strength without compromising colour stability is a desirable property for an ideal dentin bio-modifier. In this regard, riboflavin can be considered as a more effective alternative to chlorhexidine.
